# Congenital hepatic fibrosis: case report and review of literature

**DOI:** 10.11604/pamj.2021.38.188.27941

**Published:** 2021-02-18

**Authors:** Brahim El Hasbaoui, Zainab Rifai, Salahiddine Saghir, Anas Ayad, Najat Lamalmi, Rachid Abilkassem, Aomar Agadr

**Affiliations:** 1 Department of Pediatrics, Military Teaching Hospital Mohammed V, Faculty of Medicine and Pharmacy, University Mohammed V, Rabat, Morocco,; 2 Department of Pediatrics, Children’s Hospital, Faculty of Medicine and Pharmacy, University Mohammed V, Rabat, Morocco,; 3 Department of Histopathologic, Avicenne Hospital, Faculty of Medicine and Pharmacy, University Mohammed V, Rabat, Morocco

**Keywords:** Fibrosis, hyper-transaminasemia, cholestasis, ciliopathy, case report

## Abstract

Congenital hepatic fibrosis (CHF) is a rare autosomal recessive disease derived from biliary dysgenesis secondary to ductal plate malformation; it often coexists with Caroli’s disease, von Meyenburg complexes, autosomal dominant polycystic kidney disease (ADPKD), and autosomal recessive polycystic kidney disease (ARPKD). Although CHF was first named and described in detail by Kerr et al. in 1961. Its pathogenesis still remains unclear. The exact incidence and prevalence are not known, and only a few hundred patients with CHF have been reported in the literature to date. However, with the development of noninvasive diagnostic techniques such as ultrasound, computed tomography (CT), and magnetic resonance imaging (MRI), CHF may now be more frequently detected. Anatomopathological examination of liver biopsy is the gold standard in diagnosis of CHF. Patients with CHF exhibit variable clinical presentations, ranging from no symptoms to severe symptoms such as acute hepatic decompensation and even cirrhosis. The most common presentations in these patients are splenomegaly, esophageal varices, and gastrointestinal bleeding due to portal hypertension. In addition, in younger children, CHF often is accompanied by renal cysts or increased renal echogenicity. Great variability exists among the signs and symptoms of the disease from early childhood to the 5^th^ or 6^th^ decade of life, and in most patients the disorder is diagnosed during adolescence or young adulthood. Here, we present two cases of congenital hepatic fibrosis in 2-years-old girl and 12-year-old male who had been referred for evaluation of an abdominal distension with persistent hyper-transaminasemia and cholestasis, the diagnostic was made according to the results of medical imaging (CT or MRI), a liver biopsy, and genetic testing.

## Introduction

Congenital hepatic fibrosis (CHF) is a developmental disorder of the portobiliary system, characterized histologically by defective remodeling of the ductal plate (DPM). DPM refers the histologic changes seen in the liver of a heterogeneous group of genetic disorders, in which segmental dilation of the intrahepatic bile duct are associated with fibrosis. The persistence of immature embryonic bile ducts incites excess proliferation of fibrous tissue in portal areas, leading to portal hypertension, splenomegaly, hyper-splenism, upper gastrointestinal varices, and ascites. Meantime hepatomegaly is an important sign that can be detected in almost of patients; these patients have relatively well-preserved liver function [1]. Congenital hepatic fibrosis is also frequently associated with ciliopathies (disorders of primary cilia) and the phenotype may involve kidneys, collectively termed hepatorenal fibrocystic disease. Autosomal recessive polycystic kidney disease (ARPKD) is the most likely concomitant ailment, as opposed to juvenile nephronophthisis, various syndromic conditions (meckel-gruber, Bardet-biedl, jeune, or joubert), and related disorders that present with less frequency [2,3]. Here, we present two cases of congenital hepatic fibrosis according to the results of medical imaging (CT or MRI), a liver biopsy, and genetic testing.

## Patient and observation

**Case 1:** we report the 2-years-old girl who had been referred for evaluation of an abdominal distension with persistent hyper-transaminasemia and cholestasis. She was born at full term of non-consanguineous parentage with no antenatal or perinatal complications, she was on exclusively breast-fed, food diversification was started at 6 months old, the child was described as a good eater, she was on a normal diet, and was thriving appropriately. Furthermore, the girl presented an abdominal distension with recurrent episodes of mild diarrhea, bloating and loss of appetite since last 3 months. On the other side there was no history of jaundice, or gastro-intestinal bleeding. At the admission both her growth and development were normal for her age. However, hepatomegaly and splenomegaly were noted. The liver biochemical profile revealed elevated alkaline phosphatase (ALK) of 704 U/L (reference range, 38-126 U/L), alanine aminotransferase (ALT) of 264 U/L (reference range, 10-40 U/L), and aspartate aminotransferase (AST) of 153U/L (reference range, 5-34 U/L).

**Figure 1:**
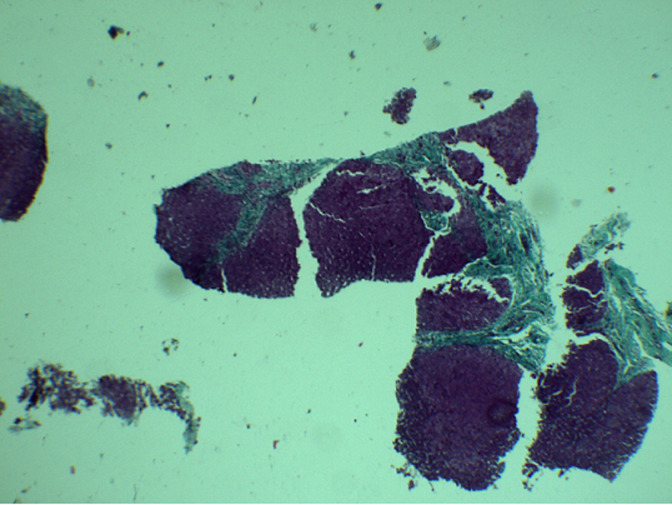
liver biopsy specimen with portal and focal periportal fibrosis at the masson’s trichrome staining (4 magnification)

**Figure 2:**
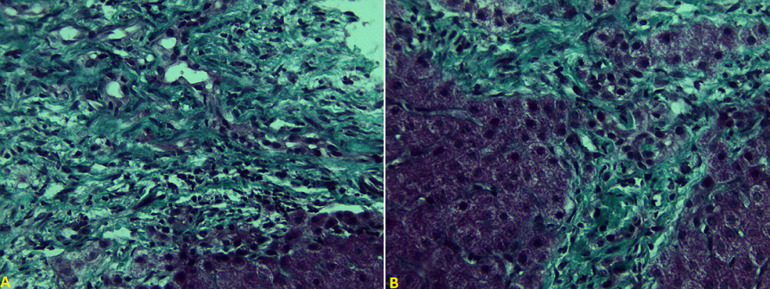
A,B) masson’s trichrome staining (40 magnification) of liver tissue showing widened portal tract with abnormally formed bile ducts and periportal fibrosis

Total and direct bilirubin, prothrombin time (PT), activated partial thromboplastin time (PTT) levels were normal. No thrombocytopenia was noticed at the blood count cell. Based on laboratory data for chronic hepatitis, further tests were performed to established etiology and included the determination of hepatitis B surface antigen (HBsAg) and hepatitis C virus (HCV), cytomegalo virus (CMV), Epstein-Barr virus (EBV) antibodies, examination of serum ceruloplasmin, 24h urine copper, serum copper, serum iron, ferritin, transferrin saturation, smooth muscle actin (SMA) antibodies, liver-kidney microsomal (LKM) antibodies, and anti-nuclear antibodies (ANA) were done and were negatives. Abdominal ultrasound showed hepatosplenomegaly with normal liver echotexture, no nodular or steatosis liver were observed, there were no signs for portal hypertension at the doppler. No fibrosis or hepatic cirrhosis, no dilatations of intrahepatic bile ducts and no renal lesions were observed in abdominal CT and MRI. On the other hand, there were no esophageal varices on upper gastrointestinal endoscopy. Liver biopsy was performed and the histopathologic exam demonstrating disordered hepatic acini and fibrous parenchymal banding bile ductular proliferation and dilation, consistent with the diagnosis of congenital hepatic fibrosis (Figure 1, Figure 2). Our patient was put on a waiting list for liver transplantation, her clinical condition remained stable, she is followed regularly, until now without any complications such as abdominal pain, jaundice or hematemesis with supportive treatment.

**Case 2:** her the second patient was a 12-year-old male referred to our hospital with hepatomegaly and splenomegaly of 2 year-duration as well as fever lasting for 3 months and abdominal distension, he had also hyper-transaminasemia and cholestasis. Like the first patient, further tests were performed to established etiology of this disorder; the results for serum ceruloplasmin, ferritin, viral markers, and antinuclear antibodies (ANA) were all within normal limits, and the T spot test was negative. However, abdominal computed tomography (CT) and magnetic resonance imaging (MRI), showed hepatomegaly, splenomegaly, dilatations of intrahepatic bile ducts but the liver parenchymal heterogeneity appeared non-cirrhotic. On the other hand, two cysts were seen in the left kidney. The histopathologic exam of liver biopsy revealed proliferation of collagen fibers surrounding the portal area, a finding that was compatible with congenital hepatic fibrosis. Like the first patient, he was discharged after his symptoms improved with supportive treatment, and he remained alive over 1 year of follow-up until he developed an acute liver and renal failure, unfortunately he died 2 months later.

## Discussion

Congenital hepatic fibrosis (CHF) is a fibrocystic disease affecting mainly the kidneys and liver. It is characterized by fusiform dilatations of the renal collecting duct and ductal plate malformation of the liver. The incidence is approximately 1: 20,000 live births. The majority of cases are caused by mutations in the PKHD1 gene, which encodes for fibrocystin/polycystin. This protein is expressed by the renal and biliary epithelium and is postulated to maintain 3-dimensional tubular architecture. Autosomal recessive polycystic kidney disease can be divided into 4 subgroups. The first group, “perinatal,” presents in infancy with severe renal disease, congestive heart failure, and pulmonary hypoplasia. The second group, “neonatal,” has progressive renal disease, whereas the third (“infantile”) and fourth (“juvenile”) groups have less severe renal disease and more complications from CHF [4,5]. Congenital hepatic fibrosis was initially reported by Bristowe in 1856. In 1961, the varied clinical findings of CHF were described by Kerr *et al*. including hepatosplenomegaly, hematemesis, and melena among 13 patients. Congenital hepatic fibrosis is usually diagnosed in early infancy or during childhood (median and mean age, 2 and 11.2 years, respectively), with the most common presentations of portal hypertension including splenomegaly and variceal bleeding [6,7]; however, some of these patients can be asymptomatic for many years, resulting in an unexpected CHF diagnosis in adulthood [8].

Ductal plate malformation, secondary to abnormal remodeling of the biliary system, appears to be the main mechanism of CHF pathogenesis. Ductal plate gives origin to the biliary system, and its defect causes excess of the embryologic bile ducts and abnormalities of portal vein branches [9,10]. The combined clinical and pathologic characteristics are largely determined by the stage of biliary malformations during fetal development. In typical CHF, the ductal plate defect is at the level of the smaller interlobular bile ducts. In contrast, the larger intrahepatic bile ducts are affected in patients with Caroli disease. Many cases with combined features of Caroli disease and CHF were reported and can be explained by ductal plate malformation affecting different segments of intrahepatic bile ducts. The associations between CHF and related ductal plate malformation entities such as Caroli disease, Von meyenburg complex (bile duct hamartoma), and choledochal cysts are well established in the literature [11]. These can drive heterogeneity in the clinical and pathologic manifestations, rendering difficulties for diagnosis. Congenital hepatic fibrosis is most commonly associated with ARPKD, which results in additional clinical ramifications and worse outcome. The onset of presentation and severity of symptoms is very variable. The majority manifests even in childhood [12]. In most patients, the first manifestations of the disease are signs and symptoms related to portal hypertension, often with gastrointestinal bleeding [13]. Hemathemesis and melena are initial symptoms in 30%-70% of cases. More rarely, they may present episodes of cholangitis. Depending on the associated diseases, patients will have signs and symptoms related to other organs such as kidneys and central nervous system (CNS) [14]. These patients classically do not have cirrhosis, maintain normal hepatic lobular architecture and liver function.

Physical examination findings include hepatomegaly, with predominant involvement of the left lobe, splenomegaly and nephromegaly. The liver is firm, with a mildly nodular surface. Laboratory workup may reveal mild elevations in liver enzymes. Patients with a predominantly cholangitic clinical picture may have marked elevations in alkaline phosphatase (ALP), γ glutamyl transpeptidase (GGT) and bilirubin. Varying cytopenias (leukopenia, thrombocytopenia) secondary to hypersplenism may be seen on a blood count. Abnormal renal functions tests are associated with extensive cystic renal disease, which may even progress to end-stage renal failure [15]. Ultrasound is usually the first method employed due to availability, low cost and harmlessness. It is able to detect alterations in the bile ducts and hepatic parenchyma, besides renal alterations. The most common findings are hypertrophy of the left lateral and caudate hepatic segments, right segment atrophy, splenomegaly, intra- and extrahepatic biliary dilatation with concomitant cystic and solid lesions (regeneration nodules), periportal thickening, hepatic and renal cysts. Computed tomography (CT) offers an advantage to us by providing a better depiction of gross morphology of the liver with accurate volume measurements and imaging of liver vasculature, as well as demonstrating any changes in the biliary tree. Periportal cuffing, indicative of the fibrotic process, may also be easily detected with CT. However, nuclear magnetic resonance imaging (MRI) with cholangioresonance is an important diagnostic alternative because it does not expose the patient to radiation and allows a very detailed evaluation of the biliary tree.

An unequivocal diagnosis of congenital hepatic fibrosis can only be made by histopathologic exam of a liver biopsy. The classical histological findings of this disorder are varying degrees of hepatic fibrosis with nodular formation, and when diagnosed late, clinical and even histological findings may be confused with liver cirrhosis. In addition, large fibrous bands are seen in the portal tract with an increased number of proliferated irregular bile ducts with normal cubic epithelium. Thus, histologic hallmarks of congenital hepatic fibrosis. These findings were seen in our patient´s liver biopsy, supporting the diagnosis. As yet, no treatment modality has been shown to actually stop or even reverse the pathological process in CHF. When considering the antifibrotic treatment, it is necessary to remember that fibrosis is a dynamic process in which the degradation of the extracellular matrix, and not only the matrix formation, is important. Moreover, many studies in the literature emphasize that liver fibrosis is a reversible process. Different researchers try to find a drug that can control the progression of the disease, stopping and even reversing fibrosis. Some drugs have shown success in animal studies, buthave failed to present benefits in humans, including colchicine, angiotensin II blocker, interferon gamma and pirfenidone [16-18]. Therefore, the therapeutic strategy for CHF, so far, is to treat the complications of the disease. Hepatic transplantation is the only curative treatment for CHF. It is indicated in the advanced stages of the disease, with development of hepatic insufficiency, or in cases of recurrent cholangitis or malignant transformation. The vast majority of cases of CHF who underwent liver transplantation, described in the literature, had Caroli´s disease associated with recurrent cholangitis. On the other hand, Geramizadeh *et al*. in 2010, reported two cases of CHF patients who required hepatic transplantation due to hepatic insufficiency, without previous or current history of cholangitis. In cases of associated renal and hepatic diseases, combined kidney and liver transplantation may be necessary. In addition, one study showed that in patients with liver and kidney disease who underwent only liver transplantation, there was an improvement in renal function [19,20].

## Conclusion

Congenital hepatic fibrosis (CHF) is a very rare disorder usually occurring in association with other fibro-polycystic disorders, including renal involvement. Thus, pure/isolated CHF is very rare. It is necessary to differentiate it from idiopathic portal hypertension and early liver cirrhosis. After a diagnosis of CHF is established, the physician must investigate other organ systems, particularly for renal involvement. A liver biopsy is essential in the diagnosis and differential diagnosis of CHF, as the presence of small bile duct dilatation and proliferation would rule out other metabolic disorders of the liver.
